# PCSK9 inhibitor alleviates experimental pulmonary fibrosis-induced pulmonary hypertension via attenuating epithelial-mesenchymal transition by suppressing Wnt/β-catenin signaling *in vivo* and *in vitro*

**DOI:** 10.3389/fmed.2024.1509168

**Published:** 2024-12-11

**Authors:** Jiancheng Lin, Zetao Pan, Jiayan Sun, Xiaowan Wang, Di Yin, Cunyang Huo, Qiang Guo

**Affiliations:** ^1^Medical College of Soochow University, Suzhou, Jiangsu, China; ^2^Department of Emergency and Critical Care Medicine, The Fourth Affiliated Hospital of Soochow University (Suzhou Dushu Lake Hospital), Suzhou, Jiangsu, China; ^3^Medical Center of Soochow University, Suzhou, Jiangsu, China; ^4^The First Affiliated Hospital of Soochow University, Suzhou, Jiangsu, China

**Keywords:** pulmonary fibrosis, pulmonary hypertension, PCSK9, EMT, Wnt, β-catenin

## Abstract

**Background:**

The co-occurrence of pulmonary hypertension (PH) in patients with pulmonary fibrosis (PF) is linked to a more unfavorable prognosis and increased mortality compared to PF cases without PH. Early intervention and comprehensive management are pivotal for improving survival outcomes. Proprotein convertase subtilisin/kexin type 9 (PCSK9) is a protein essential in cholesterol metabolism. However, the potential for PCSK9 inhibition to alleviate PF-induced PH has not been previously reported.

**Methods:**

A mouse model of PF-induced PH was established using intratracheal injection of bleomycin (BLM), followed by administration of a PCSK9 inhibitor every other day. Data on right ventricle (RV) remodeling and changes in pulmonary arteries were collected and analyzed. Transforming growth factor-beta (TGF-β) was also administered to MLE-12 cells as an experimental lung fibrosis model. The mechanisms of PCSK9’s impact on lung fibrosis were examined both *in vivo* and *in vitro*.

**Results:**

Inhibition of PCSK9 significantly reduced pulmonary artery thickening and RV remodeling in the BLM-induced mouse model. Moreover, the blockage of PCSK9 effectively attenuated the migration and epithelial-mesenchymal transition (EMT) process of TGF-β-induced MLE-12 cells. We also observed that the PCSK9 inhibitor suppressed the expression of the Wnt/β-catenin pathway in both animal and cell experiments.

**Conclusion:**

PCSK9 plays a crucial role in the progression of PF-induced PH by regulating cell EMT and Wnt/β-catenin signaling. Targeting PCSK9 expression or activity could effectively control lung fibrosis and its PH complication.

## 1 Introduction

Pulmonary hypertension (PH) is a progressive and malignant cardiopulmonary disorder characterized by excessive lung vascular remodeling, which can lead to the vascular occlusion of pulmonary small arteries (1). The World Health Organization (WHO) classifies PH into five groups based on etiology and pathophysiology. As lung fibrosis becomes increasingly prevalent in interstitial lung disease (ILD), these cardiopulmonary disorders are classified as Group III PH due to their association with pulmonary diseases (2). This association often signifies not only a high incidence but also a poor prognosis of this subtype of PH (3). The etiology and pathophysiological mechanisms of PH associated with lung fibrosis are complex, involving multiple triggers such as hypoxia-related pulmonary vasoconstriction, endothelial and smooth muscle dysfunction, inflammation, and a hypercoagulable state (4).

Pulmonary fibrosis (PF) is also an aggressive and debilitating pulmonary disease, which is recognized as the excessive deposition of fibrous substance filling in the lung parenchyma (5). This complex interplay of cellular and molecular processes results in the thickening and stiffening of lung tissue, leading to impaired gas exchange and respiratory function (6). The pathological changes in PF, primarily attributed to alveolar epithelial cell dysfunction and fibroblast activation, can cause vascular remodeling and narrowing of the pulmonary arteries and result in increased pulmonary vascular resistance and elevated pulmonary arterial pressure (7). Despite fibrotic changes being the central focus of both diseases (8, 9), the lack of effective approaches to prevent or counteract them renders PF-induced PH refractory to treatment. Current treatment strategies are insufficient and have shown limited effectiveness (10). Hence, exigent exploration is demanded to research new therapeutic angles for treating PH caused by PF.

Epithelial-mesenchymal transition (EMT), known as a pivotal cellular process during embryogenesis, tissue regeneration, and the progression of cancer and fibrosis, involves epithelial cells undergoing morphological and phenotypic changes to acquire mesenchymal cell characteristics (11). Characterized by the down-regulation of epithelial markers (e.g., E-cadherin) and the up-regulation of mesenchymal markers and EMT-specific transcription factors (e.g., Vimentin, N-cadherin, and Snail), EMT is widely regarded as an important initiator and driver of pulmonary fibrosis and its complications, where alveolar epithelial cells are exposed to profibrotic signals, such as transforming growth factor-beta (TGF-β), begin to lose their epithelial characteristics and acquire a mesenchymal phenotype (12). This phenotypic switch contributes to the pathogenesis of PF by increasing the population of fibroblasts and myofibroblasts. Fibroblasts and myofibroblasts, essential mesenchymal cells in the cellular mechanisms of PF, play key roles in the maintenance and repair of connective tissue (13). During EMT, alveolar epithelial cells transition into fibroblasts, which are then capable of producing extracellular matrix components like collagen (12). This process leads to the excessive deposition of fibrous tissue in the lung parenchyma, characteristic of PF (6). Substantial evidence indicates that EMT is governed by various signaling pathways, including the TGF-β/Smad, Wnt/β-catenin, and Notch pathways (14). These pathways are also critical in exploring the pathogenesis of PF-induced PH.

Proprotein convertase subtilisin/kexin type 9 (PCSK9) is a serine protease that plays a crucial role in cholesterol metabolism (15, 16). It is primarily known for its regulation of low-density lipoprotein receptors (LDLRs) on hepatocytes (17). PCSK9 binds to LDLRs, promoting their degradation and thereby reducing the clearance of LDL cholesterol (LDLC) from the bloodstream. Consequently, PCSK9 inhibitors, which block the interaction between PCSK9 and LDLRs, have emerged as effective therapeutic agents in lowering LDLC levels and reducing cardiovascular risk (18). Beyond its well-established role in lipid metabolism, recent research has uncovered a broader spectrum of PCSK9’s biological functions, including its involvement in inflammation, apoptosis, and various cellular signaling pathways (19, 20). Notably, PCSK9 has been implicated in the process of EMT, a critical mechanism in tissue remodeling, cancer metastasis and histological fibrosis (21).

Given the lack of research on the association among PCSK9, EMT, and PF-induced PH, we treated a bleomycin (BLM)-induced PH mouse model and recombinant transforming growth factor-β (TGF-β)-induced mouse lung epithelial (MLE-12) cells with SBC-115076, a PCSK9 inhibitor (22). This treatment revealed significant alterations in cardiopulmonary function, inflammatory response, fibrotic changes, and EMT processes. Overall, this study aims to identify a potential therapeutic target for PF-induced PH and provide experimental evidence to support it.

## 2 Materials and methods

### 2.1 Animals

Eight-week-old male C57BL/6 mice weighing approximately 23 g were obtained from Ziyuan Laboratory Animal Technology Co., Ltd. (Hangzhou, China). They were kept on a standard diet and provided with water under controlled environmental conditions: a 12-h light-dark cycle, humidity at 55 ± 5% and temperature at 23 ± 2°C. Following one-week adaptation time, 15 mice were randomly divided into three groups (*n* = 5 each) for conducting PF-induced PH models according to a previous research (23): the Control (CON) group receiving intratracheal injections of normal saline, the bleomycin (BLM)-administered group (BLM), and the BLM + SBC-115076 treated group (SBC), which received intratracheal injections of BLM (2 mg/kg, Selleck). Thirty minutes after the intratracheal injection, normal saline was intraperitoneally administered to mice in the CON and BLM groups, while those in the SBC group received SBC-115076 (10 mg/kg, Selleck). This intraperitoneal injection mode was repeated every other day for a duration of 3 weeks.

### 2.2 Right ventricular systolic pressure and right ventricular hypertrophy index measurement

On day 21, all mice underwent anesthetized with 1% isoflurane. Under ventilator anesthesia, right ventricular systolic pressure (RVSP) was measured using a microtip Millar pressure transducer catheter (SPR-839) inserted into the RV via the right external jugular vein. The curve graph depicting RVSP was continuously recorded and subsequently analyzed with the PowerLab system and LabChart software. The right ventricular hypertrophy index (RVHI), also known as the Fulton index, was evaluated as the ratio of the RV weight to the combined weight of the left ventricle (LV) and septum (S) [RV/(LV + S) ratio]. Following the surgical procedures, all mice were euthanized by cervical dislocation, and bronchoalveolar lavage fluid (BALF) along with lung tissues were collected.

### 2.3 ELISA

After RVSP measurement, BALF was collected by cannulating the trachea with a sterile catheter and instilling 1 mL of sterile phosphate-buffered saline (PBS) into the lungs, followed by gentle aspiration. This process was repeated three times, and the recovered lavage fluid was pooled for each animal. The collected BALF was then centrifuged at 1,500 rpm for 10 min at 4°C to remove cellular debris, and the supernatant was carefully separated and stored at −80°C until analysis. Levels of IL-6, TGF-β, and TNF-α in the BALF were quantified using commercially available ELISA kits (Elabscience, China) according to the manufacturer’s instructions, with absorbance measured at the appropriate wavelength using a microplate reader (Multiskan™ FC Microplate Photometer, Thermo Fisher Scientific, US).

### 2.4 Histopathology staining

The lung tissues, following fixation in 4% paraformaldehyde and subsequent embedding in paraffin, underwent sectioning into 5 μm slices. These sections were then subjected to staining using hematoxylin-eosin solution (H&E), Masson’s Trichrome stain, and Sirius Red stain kit (Servicebio, China).

### 2.5 Immunohistochemistry

The paraformaldehyde is dissolved in phosphate-buffered saline (PBS) to fix mice lung specimens at room temperature (RT) for 48 h, and subsequently embedded in paraffin. After embedding, tissues underwent sectioning into slices, deparaffinizing and rehydrating. To block endogenous peroxidase activity, 3% hydrogen peroxide was applied for 20 min at RT. Non-specific binding was then prevented by incubating the slides in 5% bovine serum albumin (BSA) for 2 h. Antibodies targeting alpha smooth muscle actin (αSMA) and collagen I (COL1A1) (Proteintech) were diluted in blocking solution and applied to the slides, which were incubated for 2 h at RT, followed by an additional hour of incubation with secondary antibodies at RT. Subsequently, diaminobenzidine (DAB) reagent and hematoxylin were used to counterstained the slides. The resulting images were captured using an ECLIPSE Ti2 inverted microscope (Nikon, Japan).

### 2.6 Cell culture

Mouse lung epithelial (MLE-12) cells were obtained from the National Collection of Authenticated Cell Cultures (Shanghai, China). The cells were maintained in Procell medium supplemented with 10% fetal bovine serum (FBS, ABW, China) and 1% penicillin-streptomycin, and cultured at 37°C in a humidified atmosphere with 5% CO2. The cells were divided into four groups: (1) the CON group, which received no special treatment; (2) the TGF-β group, where cells were treated with recombinant TGF-β (200 ng/ml); (3) the SBC group, where cells were treated with TGF-β and SBC-115076 at concentrations of 5, 10, or 20 μM; and (4) the SKL2001 group, where cells were treated with TGF-β, SBC-115076 and SKL2001 (20 μM), an agonist of the Wnt/β-catenin pathway. Phosphate-buffered saline (PBS) was used to adjust the final volumes of all solutions. Recombinant TGF-β was purchased from PeproTech (New Jersey, USA), and SKL2001 was obtained from Selleck Chemicals (Texas, USA).

### 2.7 Wound healing assay

MLE-12 cells were seeded in 6-well plates. Sterile 1,000 μl pipette tips were used to create scratches in the cell monolayer, and any dislodged cells were removed by washing with PBS. Then serum-free medium was used to substituted the normal culture medium. Initial images were captured immediately following the scratch, and again 24 h later, using a light microscope. Cell migration was quantified by converting the images to grayscale and analyzing them with ImageJ software.

### 2.8 Immunofluorescence assay

Immunofluorescence staining of MLE-12 cells was performed according to the protocol of the Immunol Fluorescence Staining Kit (Beyotime Biotechnology, China). MLE-12 cells were stained with αSMA specific rabbit polyclonal antibody and anti-COL1A1 rabbit antibody (Proteintech, China). After overnight incubation at 4°C, cells were incubated with anti-rabbit IgG conjugated secondary antibody and counterstained with DAPI (Beyotime Biotechnology, China). Fluorescent positive cells were detected using an ECLIPSE Ti2 inverted fluorescent microscope (Nikon, Japan).

### 2.9 Western blot analysis

MLE-12 cells and mouse lung tissues were lysed using lysis buffer (Thermo Fisher Scientific, USA) with phosphatase and protease inhibitors. Protein concentrations were measured using the Pierce BCA Protein Assay Kit. Equal amounts of protein (10–20 μg) were separated by SDS-PAGE (10–12%) and transferred onto polyvinylidene fluoride (PVDF) membranes. After blocking, membranes were incubated with primary antibodies against β-actin (1:7000), E-cadherin (1:20000), N-cadherin (1:7000), Vimentin (1:20000), Snail (1:700), Wnt5A/B (1:2000), and β-catenin (1:10000) (Proteintech, China), followed by secondary antibody incubation (1:10000, Proteintech). Protein bands were visualized using ECL detection reagents and quantified with ImageJ software (V1.8.0.112).

### 2.10 Statistical analysis

Differences between two groups were assessed using Student’s *t*-test with Welch correction. For multiple comparisons, one-way analysis of variance (ANOVA) followed by Bonferroni *post-hoc* test was employed. Data from a minimum of three independent experiments were analyzed using GraphPad Prism software and are presented as means ± SD. Statistical significance was defined as a *p*-value < 0.05.

## 3 Results

### 3.1 The cardiopulmonary function of mice with BLM-induced pulmonary hypertension is improved by the PCSK9 inhibitor

**FIGURE 1:**
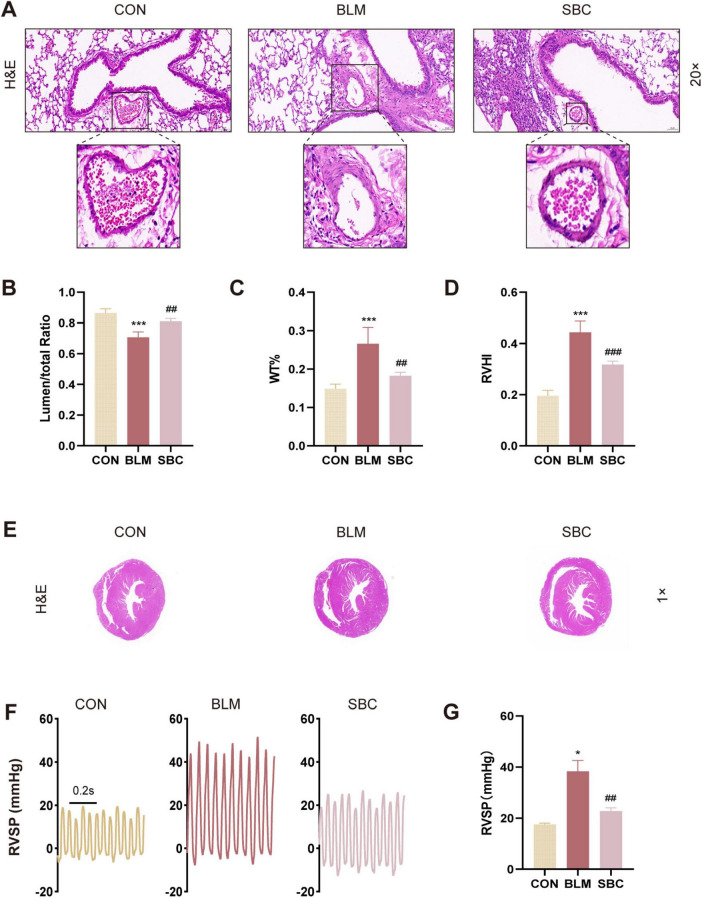
PCSK9 inhibitor ameliorates bleomycin (BLM)-induced cardiopulmonary remodeling and function **(A)** Representative images of mice lung sections stained by hematoxylin and eosin (H&E), the frame points to pulmonary arteries. **(B)** Quantitative assessment of the pulmonary artery lumen to total area ratio. **(C)** Statistical analysis of the pulmonary arterial wall thickness percentage (WT%). **(D)** Statistical analysis of the right ventricle hypertrophy index (RVHI). **(E)** RV hypertrophy shown by H&E staining. **(F)** Representative right ventricular systolic pressure (RVSP) curve. **(G)** Statistical analysis of RVSP. *p < 0.05 and ***p < 0.001 versus CON group. ^##^*p* < 0.01 and ^###^*p* < 0.001 versus BLM group.

We performed light microscopy measurements of lumen stenosis and pulmonary artery thickness to evaluate the effects of PCSK9 inhibition on pulmonary arterial reconstruction in mice induced with BLM. Histological analysis of the pulmonary arteries demonstrated a reduction in lumen area ratio, which was subsequently increased following PCSK9 inhibitor treatment. Conversely, an opposite trend was found in the pulmonary arteries using another index named the wall thickness percentage (WT%) (Figures 1A–C). These findings suggest a significant reversal of pulmonary arterial remodeling induced by PF-associated PH upon PCSK9 inhibitor administration. Notably, the observed remodeling in the pulmonary arteries was primarily confined to fibrotic lung regions, suggesting that this improvement is related to fibrosis alleviation.

Additionally, the right ventricle hypertrophy index (RVHI), also known as Fulton’s index, a parameter indicative of right ventricle hypertrophy and reconstruction, was evaluated. BLM-induced mice presented a substantial increase in RVHI compared to the control group, which was notably mitigated following PCSK9 inhibitor treatment (Figures 1D, E). We also assessed RVSP by closed-chest method to gauge hemodynamics of right ventricle. The results showed that RVSP was significantly elevated in the BLM-induced mouse model but exhibited a notable reduction post-SBC treatment compared to the control group (Figures 1F, G). These outcomes demonstrate beneficial effect of the PCSK9 inhibitor on RV remodeling and cardiopulmonary function in the PH mice model induced with BLM.

### 3.2 PCSK9 inhibitor attenuates fibrotic processes and inflammatory response in PH induced by PF

**FIGURE 2:**
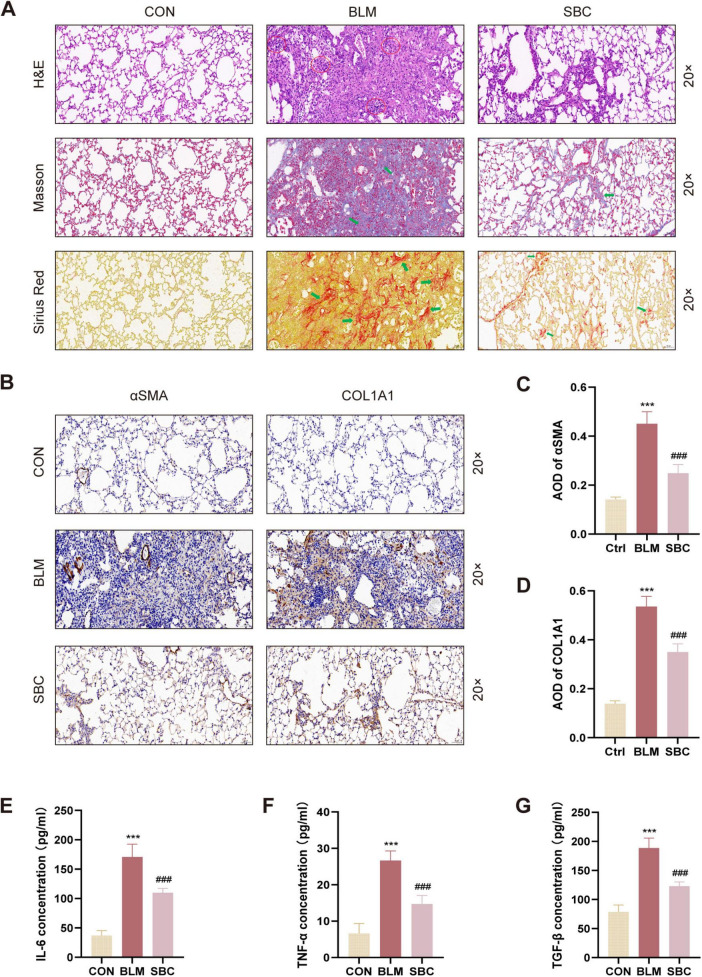
Inhibition of PCSK9 alleviates fibrotic process and inflammation response in C57BL/6 mice lung **(A)** Histological and pathological structures of lung section were shown using hematoxylin-eosin staining (H&E), Masson trichrome staining, and Sirius red staining in CON, BLM and SBC groups. Red circles indicate inflammatory cells infiltration. Green arrows indicate fibrotic deposition. **(B)** Representative immunohistochemical staining for αSMA and COL1A1. **(C)** Statistical assessment of the α-SMA and **(D)** COL1A1 positive rate. **(E)** The level of pro-inflammatory cytokines IL-6, **(F)** TNF-α and **(G)** TGF-β from bronchoalveolar lavage fluid (BALF) were quantified by ELISA. ***p < 0.001 versus CON group. ###*p* < 0.001 versus BLM group.

Histological fibrosis and alveolitis severity in the BLM-induced PH model were further evaluated by staining lung sections with H&E, Masson’s Trichrome, and Sirius Red reagents. As depicted in Figure 2A, histopathological images illustrated that sham control mice displayed normal lung architecture without collagen depositions or inflammatory cell infiltrations. BLM induction, in contrast, led to abnormal lung morphology where alveolar walls were thickened and both collagens and inflammatory cells were accumulated. Remarkably, SBC treatment mitigated these pathohistological abnormalities in lung tissues.

Furthermore, immunohistochemistry staining for αSMA and COL1A1 was conducted to comprehensively evaluate fibrotic conditions in BLM-induced lungs. As illustrated in Figures 2B–D, the average optical density (AOD) of COL1A1 and αSMA in BLM-induced lung sections significantly increased, while this metric was notably decreased in the SBC group. Additionally, as presented in Figures 2E–G, ELISA assays revealed a significant rise in the levels of inflammatory factors (IL-6, TGF-β, and TNF-α) in the BLM group’s lung tissues, which were notably suppressed after SBC treatment. These findings collectively suggest that PCSK9 inhibitor treatment ameliorated the lung architecture in BLM-induced PH by reducing collagen deposition, inflammation infiltration, and inflammatory cytokine release.

### 3.3 PF in BLM-induced mice is mitigated by PCSK9 inhibitor via reducing EMT and repressing Wnt/β-catenin signaling

**FIGURE 3:**
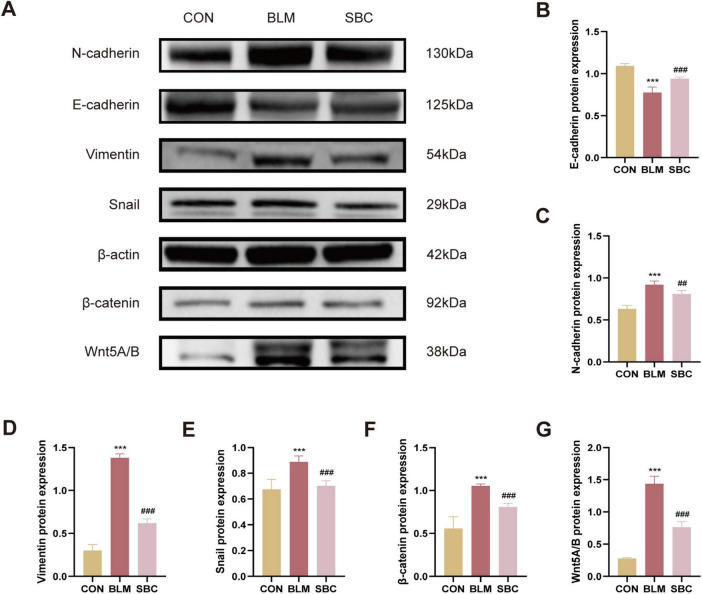
PCSK9 inhibitor regulates epithelial-mesenchymal transition (EMT) and Wnt/β-catenin pathway protein expression in C57BL/6 mice lung tissue **(A)** Typical western blot snapshots in mice lung tissue. **(B)** Quantitative analysis of the expressions of E-cadherin, **(C)** N-cadherin, **(D)** Vimentin, **(E)** Snail, **(F)** β-catenin and **(G)** Wnt5A/B in mice lung tissue. ***p < 0.001 versus CON group. ##*p* < 0.01 and ###*p* < 0.001 versus BLM group.

Protein expression analysis of biological markers associated with EMT process and Wnt/β-catenin signaling was conducted from mice lungs as well. Western blotting analysis revealed a significant increase in the synthesis levels of mesenchymal cell marker proteins including N-cadherin, Vimentin, Snail, Wnt5A/B, and β-catenin. Meanwhile, epithelial marker protein such as E-cadherin expression levels were concurrently reduced in the BLM group. Conversely, treatment with SBC exhibited the opposite trend. These findings suggest that PCSK9 inhibitor significantly attenuated the EMT process by repressing the Wnt/β-catenin signaling pathway (Figures 3A–G).

### 3.4 The migration and pulmonary fibrotic progression of MLE-12 cells were manipulated by PCSK9 inhibitor

**FIGURE 4:**
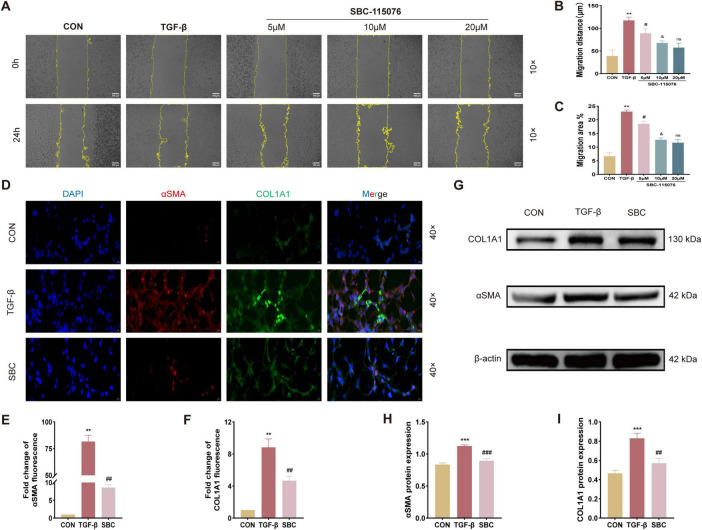
Inhibition of PCSK9 suppresses MLE-12 cell migration ability and fibrotic changes **(A)** Activity of cell migration was measured by wound healing assay and its quantified data, **(B)** migration distance and **(C)** migration area. **(D)** Representative immunofluorescent staining with αSMA and COL1A1 for MLE-12 cell. **(E)** Relative immunofluorescent intensity of αSMA and **(F)** COL1A1 in immunofluorescent images. **(G)** Typical western blot snapshots of αSMA and COL1A1. **(H)** Statistical analysis of the protein expression level of αSMA and **(I)** COL1A1 were observed and measured by western blot and its quantified data. **p < 0.01 and ***p < 0.001 versus CON group. ^#^*p* < 0.05, ^##^*p* < 0.01 and ^###^*p* < 0.001 versus TGF-β group. &*p* < 0.05 versus 5 μM SBC-115076 group. ns, no significance versus 10 μM SBC-115076 group.

To further investigate the role of PCSK9 in pulmonary fibrosis (PF), MLE-12 cells were cultured *in vitro* and treated with SBC-115076. The scratch wound healing assay (Figures 4A–C) demonstrated that TGF-β significantly enhanced the migratory capacity of MLE-12 cells. Treatment with SBC-115076 at concentrations of 5, 10, and 20 μM progressively reduced cellular migration in a dose-dependent manner. Consequently, 20 μM was selected as the optimal concentration for SBC-115076 in subsequent *in vitro* experiments to achieve the best therapeutic effect. Furthermore, immunofluorescent staining Figures 4D–F, and Western blot analysis (Figures 4G–I) of fibrosis biomarkers, including α-SMA and COL1A1, revealed that while TGF-β markedly increased their expression, the PCSK9 inhibitor effectively mitigated pulmonary fibrotic progression in MLE-12 cells.

### 3.5 SKL2001 reverses the beneficial effects of PCSK9 inhibition on EMT regulation via the Wnt/β-catenin pathway in MLE-12 cells

**FIGURE 5:**
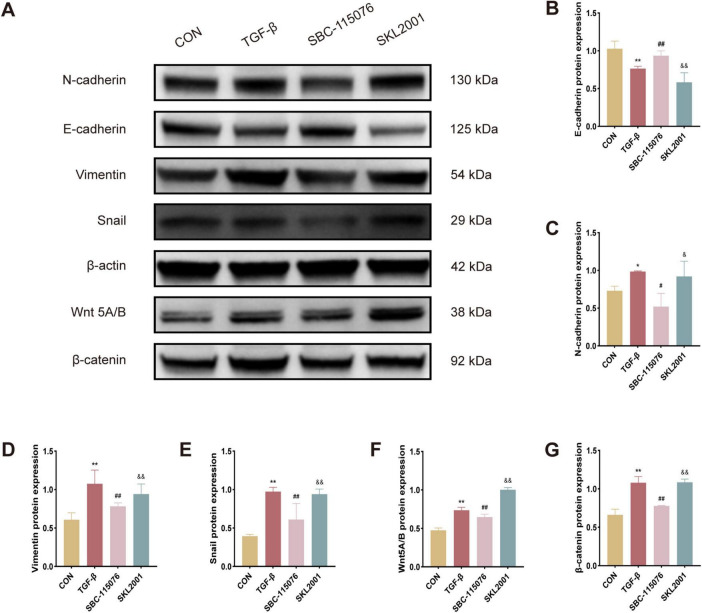
SKL2001 reverses the beneficial effects of PCSK9 inhibition on EMT regulation via the Wnt/β-catenin pathway in MLE-12 cells **(A)** Typical western blot snapshots in MLE-12 cell. **(B)** Quantitative analysis of the expressions of E-cadherin, **(C)** N-cadherin, **(D)** Vimentin, **(E)** Snail, **(F)** Wnt5A/B and **(G)** β-catanin in MLE-12 cell. *p < 0.05 and **p < 0.01 versus CON group. #*p* < 0.05 and ##*p* < 0.01 versus TGF-β group. &*p* < 0.05 and &&*p* < 0.01 versus SBC-115076 group.

In experiments involving MLE-12 cells, notable trends in EMT-related protein expression were observed among the CON, TGF-β, SBC, and SKL2001 groups, as assessed by Western blot analysis. The results showed that TGF-β treatment significantly increased the expression of EMT markers, including N-cadherin, vimentin, and Snail. Similarly, proteins associated with the Wnt/β-catenin pathway, such as Wnt5A/B and β-catenin, displayed similar upregulation. In contrast, treatment with the PCSK9 inhibitor led to a marked increase in E-cadherin expression, countering the EMT-promoting effects of TGF-β. However, SKL2001, a known agonist of the Wnt/β-catenin pathway, reversed the effects of PCSK9 inhibition on EMT-related protein expression. Collectively, these findings indicate that the PCSK9 inhibitor ameliorates cellular fibrosis in MLE-12 cells by suppressing the EMT process via the Wnt/β-catenin signaling cascade (Figures 5A–G).

## 4 Discussion

Current treatments for PH caused by PF primarily focus on supportive care, including oxygen therapy and anticoagulation, as well as antifibrotic agents (pirfenidone, nintedanib) to slow disease progression. Pulmonary vasodilators, such as endothelin receptor antagonists and phosphodiesterase-5 inhibitors, are occasionally used but have limited efficacy in Group III PH due to the underlying lung parenchymal damage. Lung transplantation remains the only curative option but is restricted by patient eligibility and donor availability (3, 4). With limited treatment options, PF is undeniably a severe and devastating disease with a disappointing survival time (6). It causes progressive fibrosis of lung tissue, obstructing oxygen distribution and exchange in the lungs, leading to breathing difficulties (7). PF can lead to PH via several mechanisms, including hypoxic vasoconstriction, mechanical compression of pulmonary vessels by fibrotic tissue, and the release of vasoactive mediators from fibrotic lungs (4, 24–26). PCSK9 inhibitors offer a novel approach by improving endothelial function, reducing oxidative stress, and mitigating inflammation. Additionally, they may indirectly suppress fibrosis progression and prevent pulmonary vascular remodeling, addressing the limitations of existing treatments (27, 28). Their multifaceted mechanism of action holds promise for comprehensive management of PF-associated PH.

Initially identified for its function in regulating LDLR degradation, PCSK9 physiologically enhances the endosomal and lysosomal degradation of hepatic LDLR, thereby increasing circulating LDL cholesterol levels (15–17). This enzyme has emerged as a significant target in cardiovascular disease therapy. Clinically, PCSK9 inhibitors, often used in conjunction with statins, have been frequently applied to reduce cardiovascular adverse events in the treatment of hypercholesterolemia (29). Shi et al. (30) confirmed that LDL-LDLR serves as an important mediator in PF, with LDLR-enhancing strategies showing beneficial effects on PF. Zhong et al. (31) also reported inflammatory response induced by LDL give rise to pulmonary artery media thickening in obese patients. Interestingly, independent of its hypolipidemic function, PCSK9 can intervene in inflammatory responses, autophagy, cell proliferation and apoptosis via various cellular pathways (19, 32–34). Specifically, PCSK9 has been implicated in promoting EMT via activating Snail 1 in cancer cells, contributing to tumor progression and metastasis (21, 35). In our study, PCSK9 inhibition conspicuously improved cardiopulmonary function, RV remodeling, and lung fibrosis progression in PH mice model induced with BLM.

EMT has been found to be widely present in multiple systemic diseases, including playing a crucial role in PF (11). The histological characteristics of PF mainly manifest as interstitial pneumonia and scattered fibroblast foci (FF) with αSMA-positive expression at collagen deposition sites (36). It has been reported that some fibroblast cells in lung fibrosis transition directly from alveolar epithelial cells (AECs) through EMT (5, 37). The interrelationship among EMT, pulmonary fibrosis, and pulmonary hypertension highlights the central role of EMT-related processes in the pathogenesis of these pulmonary diseases. In PF, EMT contributes to the fibrotic remodeling of lung tissue by generating activated fibroblasts and myofibroblasts (38). This fibrotic tissue can encroach on pulmonary vessels, leading to increased vascular resistance and the development of PH (39). Our data indicate that inhibiting PCSK9 significantly attenuated migrative ability and transition of MLE-12 cells, regulating EMT biomarker expression in both BLM-induced mouse and TGF-β-induced cell experiments.

Various factors can activate EMT in PF, including endoplasmic reticulum stress injury, TGF-β—the target of pirfenidone, an effective drug in PF treatment—and β-catenin, a crucial factor promoting pulmonary fibrosis (40–43). β-catenin induces EMT in airway epithelial cells, leading to the expression of mesenchymal markers such as αSMA and collagen I, which drive fibroblast differentiation into αSMA-positive myofibroblasts, thereby causing excessive extracellular matrix (ECM) deposition. Interestingly, emerging evidence suggests that endothelial-to-mesenchymal transition (EndMT), a variant of EMT, leads to thickening of the pulmonary arterial walls and the narrowing of the vascular lumen, contributing to the pathogenesis of PH (44). Like EMT in PF, EndMT in PH is driven by multiple signaling pathways, including TGF-β, Wnt/β-catenin, and hypoxia-inducible factors (HIFs), which are activated in response to vascular injury and hypoxia (45, 46). However, whether PCSK9 participates in EndMT remains unclear. Consistent with studies reporting enhanced Wnt/β-catenin signaling in PF models, our results demonstrate that BLM-induced animal models (*in vivo*) and TGF-β-induced models (*in vitro*) had elevated Wnt/β-catenin signaling, which was mitigated in PCSK9 inhibitor-treated groups.

In summary, this study demonstrates that PCSK9 expression induces EMT and activates the Wnt/β-catenin signaling and then enhances cell migration and invasion. PCSK9 directly or indirectly activates Snail and subsequently downregulates E-cadherin (while upregulating N-cadherin and Vimentin), inducing the EMT process. Furthermore, The PCSK9 inhibitor exerts a protective effect against BLM-induced mouse lung fibrosis and MLE-12 cell injury through modulation of Wnt/β-catenin signaling and ultimately alleviating the development of PF-induced PH. A future study examining PCSK9 as a potential therapeutic target from various perspectives for controlling PF-induced PH is anticipated.

## Data Availability

The original contributions presented in this study are included in this article/supplementary material, further inquiries can be directed to the corresponding author.
